# Impact of the COVID-19 pandemic on physical activity, smoking, alcohol use, and mental well-being—A longitudinal study of nursing students at Wroclaw Medical University in Poland

**DOI:** 10.3389/fpubh.2023.1249509

**Published:** 2023-11-16

**Authors:** Aureliusz Andrzej Kosendiak, Michał Wysocki, Paweł Krysiński, Zofia Kuźnik, Bartosz Adamczak

**Affiliations:** ^1^Department of Physical Education and Sport, Wroclaw Medical University, Wrocław, Poland; ^2^Wroclaw Medical University, Wrocław, Poland; ^3^Student Scientific Association at Department of Physical Education and Sport, Wroclaw Medical University, Wrocław, Poland

**Keywords:** physical activity, nursing students, COVID-19, cigarettes, alcohol, quality of life

## Abstract

**Introduction:**

From the moment the first cases of coronavirus disease were detected in December 2019 until the announcement and duration of the pandemic, it was a negative experience for people around the world in various spheres of life. In connection with it, there have been many changes in our daily lives related to lifestyle, physical activity, or the mental sphere. The aim of the following paper is to determine the correlation between the COVID-19 pandemic and alcohol drinking, smoking, physical exercise, and lifestyle among nursing students in Poland.

**Methods:**

The study was conducted among nursing students at Wroclaw Medical University before and during the COVID-19 pandemic. The survey consisted of completing the same anonymous online questionnaires five times by the same research group. The majority of respondents were women in the age between 18 and 30 years old and the significance level of data analysis was set at *p* < 0.05.

**Results and discussion:**

In October 2020, we recorded a large number of respondents experiencing anxiety/fear or being more stressed. Between the measurements, the highest average level of alcohol addiction (*p* < 0.001) was in October 2021 (8.71). Moreover, the percentage of respondents who felt as before increased (*p* = 0.021). As the pandemic continued, there was a systematic decline in the physical activity level (*p* < 0.001). In conclusion, the COVID-19 pandemic had a serious impact on the daily lives of the students.

## 1 Introduction

Since December 2019 the Coronavirus Disease 2019 (COVID-19) outbreak has become a devastating experience for people all over the world (1–3). Countrywide lockdown measures were taken by government authorities following the announcement of the spreading COVID-19 pandemic in March 2020 (3). A state of epidemic emergency in Poland manifested in forbidding public or state gatherings of more than 50 people, closing down shops, restaurants, and theaters, moreover, shutting down schools, universities, and other workplaces (4).

The immediate requirement of adapting to a unique reality was associated with restricting direct interpersonal contacts which expended social isolation even further (5). This extraordinary social experience had numerous negative effects on mental health (6, 7). Furthermore, the lockdown measures made physical activity less attainable to the general public, and as a result, motivation to exercise decreased (8, 9). These problems caused a shift to a more sedentary lifestyle (10). As recent studies suggest, lifestyle changes could have also had an additional impact on increased stress levels, anxiety, and depression (11–13). Moreover, stress is strongly correlated with alcohol consumption (14) and could encourage smoking (15). Healthcare professionals are particularly vulnerable to infection, including COVID-19, and therefore to greater stress (16).

Many students developed unhealthy mechanisms during the pandemic to cope with their elevated sense of anxiety, stress, or depression. In many cases this leads to increased addictive behaviors and decreased frequency of physical activity (16–18), which are some of the underlying causes of the diseases of affluence (19, 20). Besides a negative effect on all physical activity attempts, the changes affected adverse eating habits as well (21). Additionally, it was examined that in some research groups consisting of nursing students almost half of them had gained weight and had not exercised regularly (22). Moreover, recent research revealed the increasing rate of misuse of alcohol during the lockdown period amongst students (23), as well as increased tobacco use (24). These tendencies in quarantine conditions were also observed specifically amongst nursing students (25, 26). Other differences in the students' daily routine were connected with sleep disturbances and sexual dysfunctions (27), which can cause deterioration in well-being. The above data clearly indicate the negative changes taking place in the lives of students. One might suspect that there are ways to reduce the severity of these alterations or eliminate them completely.

Most studies conducted among students did not test COVID-19's effect directly on the group of nursing students (16–21, 23, 24, 27). Moreover, they referred to specific issues. Another limitation of the available literature was the short period of impact study during the pandemic (22, 25, 26). Therefore, we choose to collate and examine how levels of physical activity, alcohol abuse, nicotine addiction, and overall psychological well-being of nursing students differed over the course of the COVID-19 pandemic. Due to legal restrictions, expected was a decrease in physical activity. Moreover, we predicted an increase in negative emotions, resulting from the uncertainty of the development of the events, fear for the health of oneself and loved ones, restriction of social contacts, and reduced opportunities for relaxation. Additionally, an increase in substance abuse was expected. An important advantage of our study was focusing not only on the pre-pandemic period and the first wave of infections, but also on monitoring parameters during the subsequent stages of the pandemic. This allowed for the collection of more comprehensive data. Examining the aforementioned factors is highly relevant in this particular research group as its planned profession will require high resistance to stress and the negative effects of isolation during long-hour hospital shifts (28). Furthermore, it will be expected of them to promote a healthy lifestyle amongst their patients, as they perform a significant social function (29). Hence, it is crucial to monitor risky behaviors, lifestyle habits, and different coping mechanisms of medical-related degree students. Learning about the mechanisms behind the negative changes in the lives of future medics can make it possible to better prevent or combat them, and therefore build a more solid foundation for a resilient healthcare system.

## 2 Materials and methods

### 2.1 Study design and participants

The study was conducted in accordance with the Declaration of Helsinki, and approved by the Institutional Review Board (or Ethics Committee) of the Bioethics Committee at the Medical University of Wroclaw (no. KB-251/2020). The study was conducted among nursing students at the Wroclaw Medical University, participating in Physical Education classes during their study course. All respondents were informed of their anonymity and their answers were used only in the research. The attendees gave their informed consent to participate in the study. Inclusion criteria were the status of the nursing student at the Wroclaw Medical University, age 18–30, absence of chronic disease, and consent for research. On the other hand, exclusion criteria were no status of the nursing student of the Wroclaw Medical University, undergoing chronic illnesses during the study, and no consent for research. Students selected as eligible were invited to participate in the research. Reminders and links to the online questionnaire were sent to them via university e-mail during each data collection period. Study participants had 4 weeks to complete them every time.

### 2.2 Outcome measures

**Figure 1:**
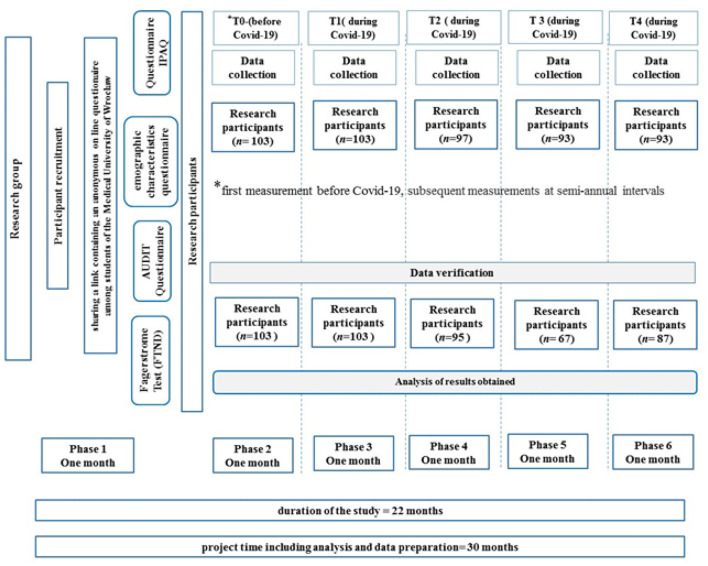
Selection process of the study. AUDIT, Alcohol Use Disorders Identification Test; n, a number of participants; T0, T1, T2, T3, T4, measurement periods; phase 1,2,3,4,5,6, stages of the research project.

The questionnaire consisted of the Fagerström Test for Nicotine Dependence (FTND), The Alcohol Use Disorders Identification Test (AUDIT), and International Physical Activity Questionnaire (IPAQ), and the demographic characteristics questionnaire, which included questions about the anthropometric data and the COVID-19 pandemic. The research was conducted in the period from October 2019 to October 2021, resulting in 5 measurements. The data collection process involved administering questionnaires through sociological investigation, which is a standard quantitative research method in public health sciences. All 1st year nursing students were selected and the study was continued in the same research group. The first survey was conducted in October 2019, 4 months before the beginning of the COVID-19 pandemic (T0). The second measurement was conducted at the beginning of the COVID-19 pandemic in Poland—March 2020 (T1). The third measurement was conducted 6 months later—in October 2020 (T2). The fourth measurement was conducted after 1 year of the pandemic—in March 2021 (T3). The last measurement was conducted in October 2021 (T4). In total 455 responses were included in the study during the whole collection period. Responses in which participants failed to complete or returned incomplete questionnaires were excluded from the study. The non-compliant responses in verification questions were eliminated as well. The entire process of preparation and selection of study participants for the research are depicted in Figure 1.

### 2.3 Demographic characteristics questionnaire

The study used a self-administered questionnaire, which included questions on age, gender, and place of residence. It also contained anthropometric data such as weight and height, from which the Body Mass Index was calculated according to accepted criteria. Moreover, the self-administered questionnaire included questions about the COVID-19 pandemic, containing the restrictions applied, its impact on the subjects, and their well-being during the pandemic. These questions did not appear until the second measurement, i.e. March 2020, when the COVID-19 Pandemic broke out. Most of the questions were closed with single or multiple choice answers. However, in some cases the original scale, e.g. Likert, was used. The answers to these questions were the subjective assessment of the participants. The reliability of the COVID-19 metric and related questions was calculated using the Cronbach-Alpha test (30).

### 2.4 Level of physical activity

To estimate the physical activity level we used the Short Polish version of the International Physical Activity Questionnaire (IPAQ-SF). It consists of 7 questions regarding the amount of time spent on various types of physical activity “during the last 7 days”. As the study shows, the completion of the questionnaire by respondents may exaggerate the results; therefore, all participants of the survey were trained by our research team (31). The results of the study were presented in energy expenditure expressed in Metabolic Equivalent of Work (MET) units (consumption of 3.5 mL of oxygen per kilogram of body weight per minute). Most of the data, unless noted otherwise, were given in minutes a week (m/w). The questionnaire divided the activities according to the following values: for walking MET = 3.3 (walkMET); for moderate intensity MET = 4 (ModMET); for vigorous activities MET = 8 (VigMET). Then we divided respondents into 3 categories: health-enhancing physical activity (HEPA)—at a minimum of 3,000 MET m/w; Minimally active—at 600–3,000 MET m/w; Inactive—below 600 MET m/w (32). The validity and reliability of the above questionnaire were confirmed experimentally (33).

### 2.5 The AUDIT test

Alcohol use disorders were diagnosed with the World Health Organization's AUDIT Test (34). It consists of three parts related to the amount and frequency of drinking (questions no. 1–3), alcohol dependence (questions no. 4–6), and four questions on problems caused by alcohol (questions no. 7–10). The questionnaire divided the respondents according to the following values: 8–15 points indicate probable hazardous drinking, 16–19 points indicate harmful drinking, and 20 points or more probable alcohol dependence. The validity and reliability of the above questionnaire were confirmed experimentally (35).

### 2.6 The Fagerström test

The probability of whether a person is pharmacologically addicted to nicotine was assessed using the international Fagerström questionnaire (36–38). It consists of six questions, each of which ranges from 0 to 1 or 0 to 3. In order to assess the level of addiction to nicotine, all the results are summed up. Obtaining 0–4 points indicates no pharmacological addiction to nicotine or a very “low” level—rather, smoking is a habit that a person does not want or cannot get rid of. Obtaining 5–8 points indicates the presence of features of nicotine addiction—it is difficult for such a person to do without a cigarette, especially in stressful situations or following an example (under pressure, at the instigation of the environment). Obtaining 9–11 points clearly indicates the presence of symptoms of pharmacological nicotine addiction and some illnesses and discomforts that are undoubtedly related to smoking. The validity and reliability of the above questionnaire were confirmed experimentally (39, 40).

### 2.7 Statistical analysis

The data were analyzed and counted with Microsoft Excel and Statistica 13.3 (Wroclaw Medical University's license, Statsoft Polska Sp. z o.o.) package. All of the personal data were combined and provided in tables. Anthropometric data were calculated and presented as mean value and standard deviation (SD). Data from the place of residence were divided into six parts, depending on the population of the city. The study was conducted among nursing students, the majority of whom are women. As a result, data analysis of differences between genders was not performed because the results would be unreliable. The ANOVA test was used to compare sets of numerical data (2nd COVID-associated question, MET, AUDIT, Fagerstrom). The χ2 (chi-square) test of independence was performed to assess statistically significant differences between expected and observed values contained in a contingency table (1st COVID-associated question, IPAQ, Place of residence, Barriers and Motives). It was also used to check the dependence of all measured values throughout the study. The impact of the physical activity level on alcohol consumption was also calculated using the χ2 test. COVID-associated questions have been grouped and shown as percentages among respondents. The variances of the single samples were compared using the F-test. Data of body weight, BMI, walking, and sitting time are normally distributed and were calculated using T-test. The significance level of data analysis was set at *p* < 0.05. When the result was statistically significant, a *post-hoc* test (Bonferroni correction) was performed to compare each measurement. We have given each comparison the appropriate letter designation: T0 vs. T1 (a), T0 vs. T2 (b), T0 vs. T3 (c), T0 vs. T4 (d), T1 vs. T2 (e), T1 vs. T3 (f), T1 vs. T4 (g), T2 vs. T3 (h), T2 vs. T4 (i), T3 vs. T4 (j).

## 3 Results

### 3.1 Basic information

The great majority of our research group were women (from 85.4 to 96.6%). When it comes to the average age it oscillates between 20.0 and 21.6 years. Interesting results were recorded in the place of residence (*p* < 0.001). Between T0 and T1, the respondents headed to the countryside. Moreover, we found a decrease in the number of cities' dwellers—except for cities with 5–20 thousand inhabitants. In the following surveys, there was a gradual decrease in the number of people living in the countryside and an increase in cities. In T4 there was the highest percentage of people living in cities over 50 thousand inhabitants (Table 1). In the *post-hoc* test, insignificant changes were only between measurements T0 and T2 (b), T0 and T3 (c), T2 and T3 (h) (Table 5).

**Table 1:** Anthropometric data and place of residence.

	**October 2019 (T0)**	**March 2020 (T1)**	**October 2020 (T2)**	**March 2021 (T3)**	**October 2021 (T4)**	***p-*value**
Female (%)	88 (85.4%)	88 (85.4%)	88 (92.6%)	60 (89.6%)	84 (96.6%)	N.A.
Male (%)	15 (14.6%)	15 (14.6%)	7 (7.4%)	7 (10.4%)	3 (3.4%)	
Age (years) mean ± SD	20.0 ± 3.1	20.3 ± 3.1	20.5 ± 2.7	21.2 ± 3.1	21.6 ± 1.0	N.A.
Height (cm) mean ± SD	168.4 ± 8.0	168.5 ± 8.0	167.3 ± 7.1	167.2 ± 6.9	166.1 ± 6.1	N.A.
Weight (kg) mean ± SD	62.9 ± 12.4	63.1 ± 12.2	61.7 ± 10.8	63.8 ± 11.2	60.9 ± 8.3	0.467
**Body mass index amount (%)**						
<18.5 Underweight 18.5–24.9	10 (9.7%)	8 (7.8%)	10 (10.5%)	3 (4.5%)	7 (8.0%)	0.558
Normal	79 (76.7%)	80 (77.7%)	75 (78.9%)	47 (70.1%)	69 (79.3%)	
25.0–29.9 Overweight	12 (11.7%)	13 (12.6%)	7 (7.4%)	13 (19.4%)	8 (9.2%)	
>29.9 Obese	2 (1.9%)	2 (1.9%)	3 (3.2%)	4 (6.0%)	3 (3.4%)	
**Place of residence amount (%)**						
Village	29 (28.4%)	40 (38.8%)	32 (33.7%)	22 (32.8%)	21 (24.1%)	<0.001^*^
Town 5,000–20,000	9 (8.8%)	17 (16.5%)	9 (9.5%)	5 (7.5%)	6 (6.9%)	
Town 20,000–50,000	15 (14.7%)	9 (8.7%)	11 (11.6%)	8 (11.9%)	15 (17.2%)	
City 50,000–100,000	12 (11.8%)	11 (10.7%)	11 (11.6%)	7 (10.4%)	11 (12.6%)	
City 100,000–500,000	13 (12.7%)	10 (9.7%)	8 (8.4%)	5 (7.5%)	34 (39.1%)	
City >500,000	25 (24.5%)	16 (15.5%)	24 (25.3%)	20 (29.9%)	0 (0%)	

N.A., not available to calculate.

^*^a,d,e,f,g,i,j, statistically significant in *post-hoc* test (for details, see Table 5).

### 3.2 Body weight and BMI

Body Mass Index was calculated based on data from WHO (World Health Organization), but for the purposes of the study, respondents with a value above 30 were combined into one group. Comparing the period before the beginning and four periods during the pandemic we did not note statistically significant changes in the body weight measurements (*p* = 0.467) and the BMI values (*p* = 0.558). The correct BMI is in the range of 70–80% of the respondents in each measurement. Underweight oscillates between 4.5–10.5%, overweight in 7.4–19.4%, and obesity from 1.9% to 6% of respondents (Table 1).

### 3.3 The pandemic impact on mental health

**Figure 2:**
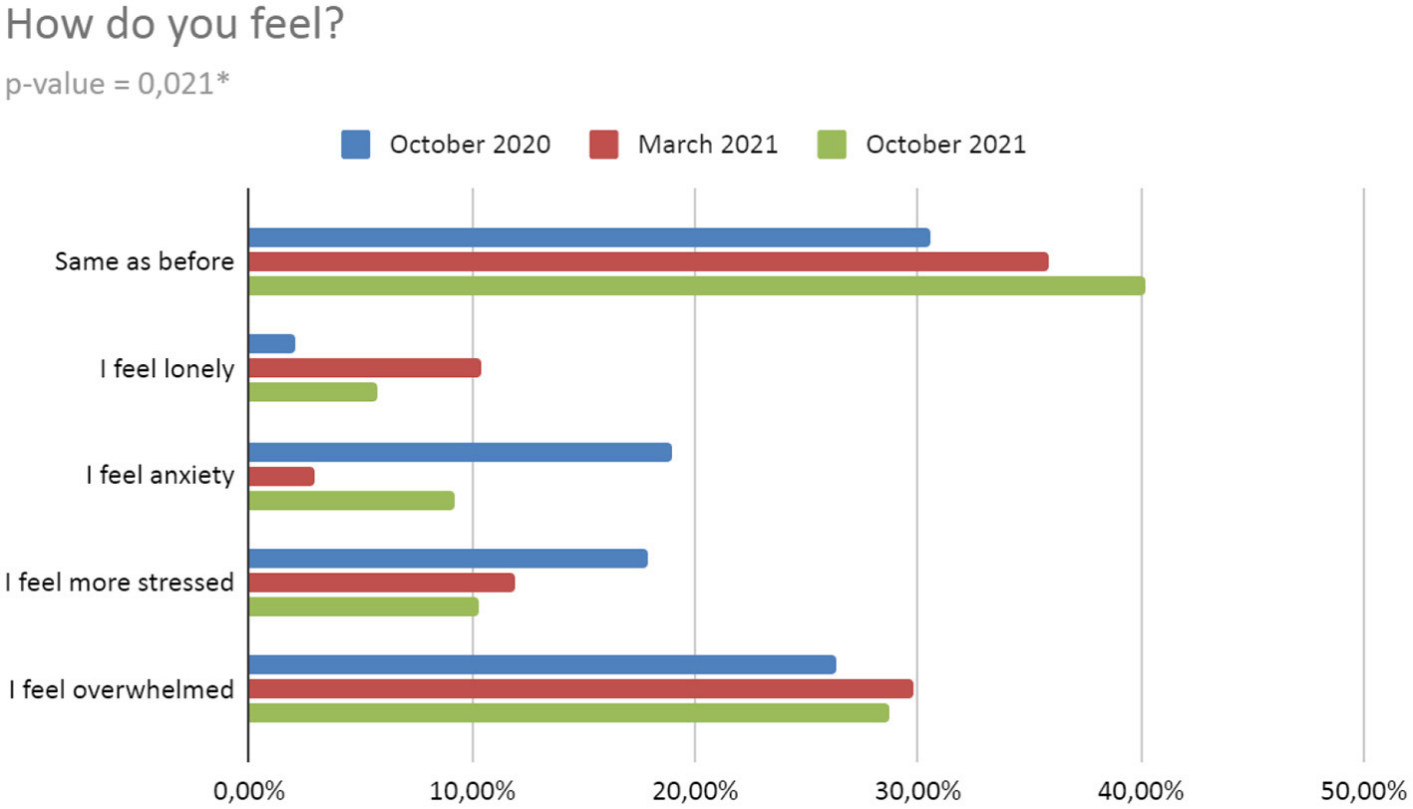
Well-being during the Pandemic. *h, statistically significant in *post-hoc* test (for details, see Table 5).

Significant differences occurred in the responses about the well-being of the respondents depending on the time of the survey (*p* = 0,021). The respondents described their condition more and more often as the same as before pandemic COVID-19: T2−30.53%; T3−35.82%; T4−40.23%. In every survey, 25–30% of students were overwhelmed by the situation, and in T2, as many as 18.95% of respondents felt anxiety/fear for themselves and their relatives. In T3 it was only 2.99%, while in T4 it was 9.20%. Moreover, in T2, 17.89% of respondents felt more stressed. In T3 it was 11.94%, and in T4 it was 10.34% (Figure 2). In the *post-hoc* test, we noted significant changes only between measurements T2 and T3 (h) (Table 5).

**Figure 3:**
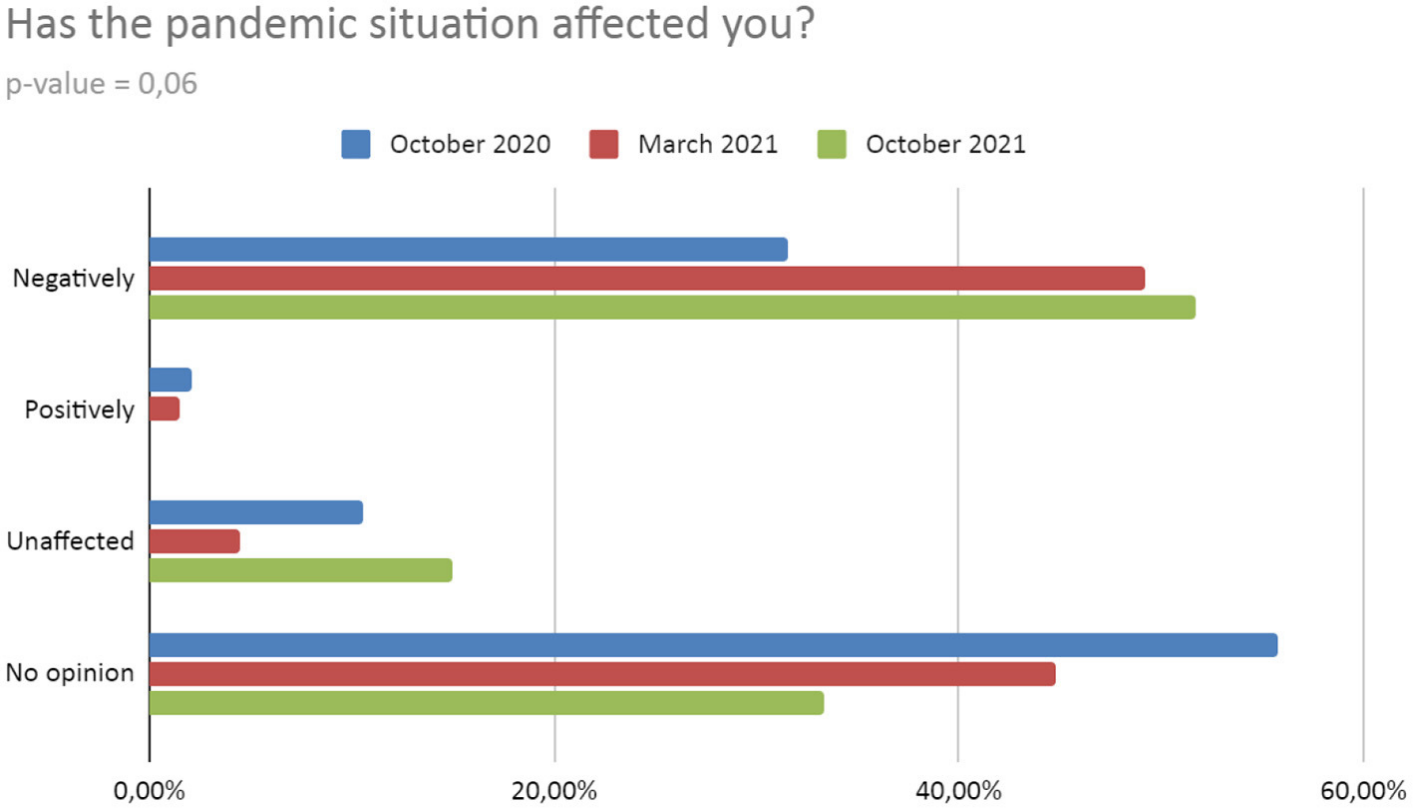
Impact of pandemic on well-being.

Changes close to statistical significance occurred in the responses about the direct pandemic impact. With each survey, an increasing number of respondents rate the impact of the pandemic as negative. A very small percentage of students assess the impact of the pandemic as positive and it decreases with each subsequent survey. The pandemic situation did not affect the following percent of respondents: T2−10.53%; T3−4.48%; T4−14.94%. The opinion related to the pandemic situation assessment was not expressed by the percentage of respondents: T2−55.79%; T3−44.78%; T4−33.33% (Figure 3).

### 3.4 Level of physical activity

When it comes to physical activity, there are statistically significant differences in the level of IPAQ depending on the time of the survey (*p* < 0,001). First of all, a systematic decrease in the number of people with high IPAQ was noticed- from 29.1% in T0 to 6.9% in T4. Simultaneously, there was an increase in the number of people with low IPAQ- from 64.1% in T0 to 86.2% in T4. While the percentage of respondents with average IPAQ remained quite constant: T0−6.8%; T1−17.5%; T2−3.2%; T3−6.0%; T4−6.9% (Table 2). In the *post-hoc* test insignificant changes were only between measurements T2 and T3 (h), T3 and T4 (j) (Table 5). Moreover, there are also statistically significant changes in MET (*p* < 0.001). The highest average MET was in T0- 4591 and the lowest in T1 (lockdown)- 1783. However, there was an increase in T2 and a systematic decrease in subsequent surveys. Except for March 2020, where VigMET has the largest share of MET, WalkMET was dominant. In the next survey, a downward trend was observed in VigMET, WalkMET, and ModMET. The results of the *post-hoc* test are presented in Table 5.

**Table 2:** Level of physical activity.

	**October 2019 (T0)**	**March 2020 (T1)**	**October 2020 (T2)**	**March 2021 (T3)**	**October 2021 (T4)**	***p-*value**
**IPAQ level**						
Low	66 (64.1%)	66 (64.1%)	75 (78.9%)	55 (82.1%)	75 (86.2%)	<0.001^*^
Medium	7 (6.8%)	18 (17.5%)	3 (3.2%)	4 (6.0%)	6 (6.9%)	
High	30 (29.1%)	19 (18.4%)	17 (17.9%)	8 (11.9%)	6 (6.9%)	
How much time did you average spend walking during the day? (minutes) (Mean ± SD)	125 ± 160	29 ± 94	104 ± 98	106 ± 113	63 ± 79	<0.001^**^
How much time did you average spend sitting during the day? (minutes) (Mean ± SD)	236 ± 240	137 ± 196	307 ± 195	366 ± 289	1,040 ± 1,008	<0.001^**^

^*^a-g,i - statistically significant in *post-hoc* test (for details, see Table 5).

^**^a,c-g,i,j - statistically significant in *post-hoc* test (for details, see Table 5).

We noted statistically significant differences also in the time spent sitting among the respondents (*p* < 0.001). The lowest average time was at the beginning of the pandemic in T1. However, the highest in the last study in T4. Moreover, an upward trend was observed from the first lockdown. There are statistically significant changes in the time spent walking too (*p* < 0.001). The highest average was in T0 and the lowest average in T1−29.1 minutes (Table 2). This is correlated with the WalkMET results. The results of the *post-hoc* test are presented in Table 5.

### 3.5 Exercise barriers and motives

There are very strong statistically significant differences in the exercise barriers (*p* < 0.001). “No barriers” were reported from 2.1% T2 to 25.2% of respondents in T1 (lockdown). The most common barriers are “lack of motivation” and “lack of time”. “Lack of time” was extremely low T1 (3.9%), in the remaining surveys it oscillated between 53.7–67.8% of students. However, in T1 the following barriers are much more common: “lack of appropriate equipment” (28.2%), and “applicable state regulations” (35.9%). Health causes as a barrier was the lowest in T1, and the highest in T3 (Table 3). The results of the *post-hoc* test are statistically significant in every measurement comparison (Table 5).

**Table 3:** Assessment of barriers (*p* < 0.001).

**Barriers**	**October 2019 (T0)**	**March 2020 (T1)**	**October 2020 (T3)**	**March 2021 (T4)**	**October 2021 (T5)**	***p-*value**
None	15.5%	25.2%	2.1%	4.5%	5.7%	<0.001^*^
Lack of motivation	30.1%	40.8%	60.0%	61.2%	51.7%	
Lack of time	54.4%	3.9%	67.4%	53.7%	67.8%	
Health reasons	11.7%	1.9%	14.7%	22.4%	13.8%	
Lack of appropriate equipment	0.0%	28.2%	0.0%	11.9%	0.0%	
Applicable state regulations	0.0%	35.9%	0.0%	1.5%	0.0%	

^*^All measurement comparisons were significant in the *post-hoc* test (Table 5).

**Figure 4:**
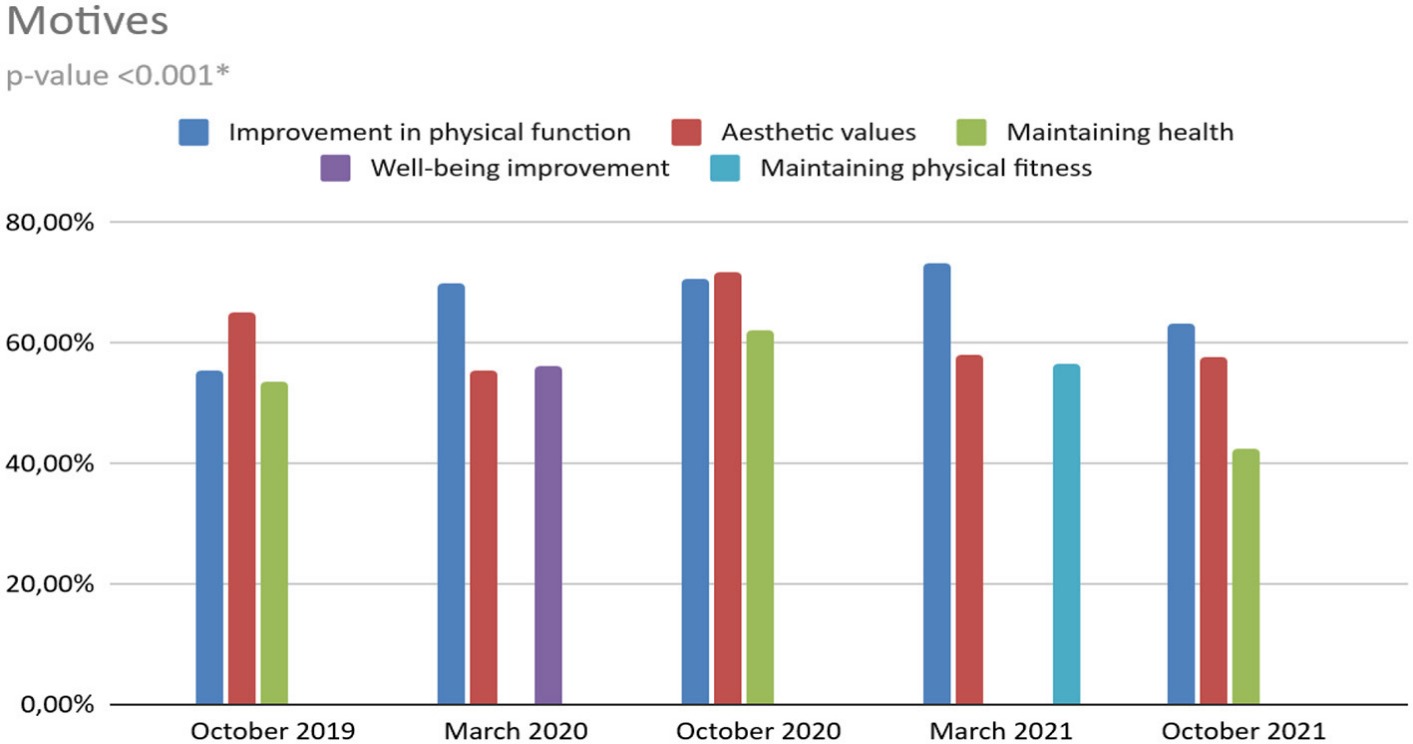
Assessment of motives (*p* < 0.001). *All measurement comparisons were significant in the *post-hoc* test (for details, see Table 5).

There are also statistically significant differences in exercise motives (*p* < 0.001). By far the most common motives are: “improvement in physical function”, “aesthetic values”, “improving or maintaining health” and “maintaining physical fitness”. An “improvement in well-being” in T1 was indicated by 56.3% of the respondents. However, in the remaining surveys it was already 0%. “Immunity improvement” was popular in T1 (20.4%) and T3 (34.3%). In the remaining studies, it was not chosen by anyone. The “fight against depression” from T0 to T3 oscillates between 8–10%. It is worth noting that in T4 it is as much as 21.8%. “Fighting addictions” was chosen by 7.8% of students. During the first lockdown in T1, it was only 1%, then it systematically grew up to 4.6% in T4. In addition, the desire to test yourself oscillates between 13.7% and 16.4%, while in T1 it is only 5.8% (Figure 4). The results of the *post-hoc* test are statistically significant in every measurement comparison (Table 5).

### 3.6 Alcohol and cigarettes

Comparing the period before the beginning and four periods during the pandemic we did not note statistically significant changes in the degree of nicotine addiction among respondents (p = 0.224). What is worth mentioning, there were no people with high and very high addiction in T2, while in T4- 4 people with high and 2 people with very high addiction (Table 4).

**Table 4:** Assessment of nicotine and alcohol addiction.

	**October 2019 (T0)**	**March 2020 (T1)**	**October 2020 (T2)**	**March 2021 (T3)**	**October 2021 (T4)**	***p-*value**
**Nicotine addiction**						
Very Low	98 (95%)	90 (87%)	93 (98%)	63 (94%)	73 (84%)	0.224
Low	4 (4%)	8 (8%)	1 (1%)	3 (4%)	5 (6%)	
Medium	0 (0%)	1 (1%)	1 (1%)	0 (0%)	3 (3%)	
High	1 (1%)	2 (2%)	0 (0%)	1 (1%)	4 (5%)	
Very high	0 (0%)	2 (2%)	0 (0%)	0 (0%)	2 (2%)	
**Alcohol addiction**						
Very low	67 (65%)	98 (95%)	73 (77%)	61 (91%)	26 (30%)	<0.001^*^
Low	31 (30%)	5 (5%)	21 (22%)	6 (9%)	57 (66%)	
Medium	4 (4%)	0 (0%)	1 (1%)	0 (0%)	3 (3%)	
High	1 (1%)	0 (0%)	0 (0%)	0 (0%)	1 (1%)	

^*^a,c-j - statistically significant in *post-hoc* test (for details, see Table 5).

On the other hand, we observed statistically significant differences in the degree of alcohol addiction (*p* < 0,001). The lowest average level of addiction was in T1—no people with a high probability of being addicted or with a suspicion of addiction, while the highest was in T4 (8.71). Moreover, risky alcohol consumption was significantly the most frequent in T4 and average alcohol addiction in studies conducted in October was 2–4 times higher than those performed in March (Table 4). In the *post-hoc* test no statistically important changes were only between measurements T0 and T2 (Table 5).

**Table 5:** *Post-hoc* test (Bonferroni correction).

	**T0 vs. T1 (a)**	**T0 vs. T2 (b)**	**T0 vs. T3 (c)**	**T0 vs. T4 (d)**	**T1 vs. T2 (e)**	**T1 vs. T3 (f)**	**T1 vs. T4 (g)**	**T2 vs. T3 (h)**	**T2 vs. T4 (i)**	**T3 vs. T4 (j)**	**Bonferroni correction**
Place of residence *P-*value significant	0.004 Yes	0.131 No	0.026 No	0.001 Yes	0.001 Yes	0 Yes	0 Yes	0.009 No	0 Yes	0 Yes	<0.005
Well-being *P-*value significant	N.A.	N.A.	N.A.	N.A.	N.A.	N.A.	N.A.	0.003 Yes	0.015 No	0.066 No	<0.0167
Level of physical activity *P-*value significant	0.001 Yes	0.002 Yes	0.003 Yes	0.001 Yes	0.001 Yes	0.001 Yes	0.001 Yes	0.06 No	0.004 Yes	0.007 No	<0.005
Average MET *P-*value significant	0.001 Yes	0.025 No	<0.001 Yes	<0.001 Yes	<0.001 Yes	0.001 Yes	0.483 No	0.020 No	<0.001 Yes	0.001 Yes	<0.005
Sitting time *P-*value significant	0.001 Yes	0.011 No	<0.001 Yes	<0.001 Yes	<0.001 Yes	<0.001 Yes	<0.001 Yes	0.015 No	<0.001 Yes	<0.001 Yes	<0.005
Walking time *P-*value significant	0.001 Yes	0.011 No	<0.001 Yes	<0.001 Yes	<0.001 Yes	<0.001 Yes	<0.001 Yes	0.015 No	<0.001 Yes	<0.001 Yes	<0.005
Barriers *P-*value significant	<0.001 Yes	<0.001 Yes	<0.001 Yes	<0.001 Yes	<0.001 Yes	<0.001 Yes	<0.001 Yes	<0.001 Yes	<0.001 Yes	<0.001 Yes	<0.005
Motives *P-*value significant	<0.001 Yes	<0.001 Yes	<0.001 Yes	<0.001 Yes	<0.001 Yes	<0.001 Yes	<0.001 Yes	<0.001 Yes	<0.001 Yes	<0.001 Yes	<0.005
Alcohol addiction *P-*value significant	<0.001 Yes	0.104 No	0.001 Yes	<0.001 Yes	<0.001 Yes	<0.001 Yes	<0.001 Yes	0.002 Yes	<0.001 Yes	<0.001 Yes	<0.005

N.A., not available to calculate.

On the other hand, we observed no influence of physical activity (IPAQ) on alcohol addiction (Table 6).

**Table 6:** Effect of IPAQ on alcohol addiction-insignificant result.

**Effect of physical activity (IPAQ) on alcohol addiction**	**October 2019 (T0)**	**March 2020 (T1)**	**October 2020 (T2)**	**March 2021 (T3)**	**October 2021 (T4)**
χ^2^	2.694	0.04	1.756	1.577	5.787
df	6	6	6	6	6
*p-*value	0.846	1	0.941	0.954	0.447

χ^2^, Chi-square; df, degrees of freedom.

## 4 Discussion

### 4.1 Anthropometric data and place of residence

The COVID-19 pandemic affected people in many ways, including their weight. As indicated by a study conducted on the Polish population by Sidor, almost 30% of respondents experienced weight gain (mean 3.0 kg). On the other hand, over 18% of people reported weight loss (mean 2.9 kg) (41). It means that nearly half of the respondents reported a weight change during the pandemic, with a majority of people with weight gain. On the other hand, data from a university in Spain indicate that the weight is maintained or increased by an average of nearly 1 kg. However, Sanchez, in a study on the Spanish population, reported an increase in weight in 52.7% of respondents, the majority of which is an increase of 1–3 kg (42).

In contrast to the above studies, we did not obtain any statistically significant changes between measurements in both weight and BMI. The difference in the average weight of the respondents between the measurements never exceeded 3 kg. The correct BMI was in the range of 70–80% of the respondents in each measurement. Underweight oscillated between 4.5–10.5%, overweight between 7.4–19.4%, and obesity between 1.9–6%. Comparing the percentage of respondents with normal BMI from our study with the population studies, the data looks positive. Compared to the data from Spain, this percentage is higher by 22.3–38.4%, from Chile by 50.0–66.2%, and from the Netherlands by 57.9–78.6% (42–44). However, compared to data from a university in Spain, the percentage is similar (45). In the case of BMI, we also did not obtain statistically significant results. Both weight and BMI values were sinusoidal and there was no clear trend. Therefore, we reject our working hypothesis of the negative impact of the COVID-19 pandemic on BMI and weight. A possible explanation for the lack of significant differences between weight measurements and BMI in this study, compared to the aforementioned one, could be due to the specificity of the group—young nursing students. This group may be more motivated to maintain a healthy weight (as a result of a greater concern for their appearance and professional association with medicine, thus better awareness of the risks posed by obesity) and may find it easier to lose it if gained (due to their greater vigor and higher metabolic rate, compared to an older population) (46).

Interesting results were recorded in the place of residence. The project of the Government Population Council on the demographic situation in Poland shows that in 2020 the trend from previous years was maintained (47). The number of inhabitants of villages and the largest urban centers with more than 500 thousand increased, while the number of inhabitants of smaller cities decreased. On the other hand, our work shows that the surveyed students in T1 headed to the countryside, but we find a decrease in the number of cities dwellers—except for cities 5–20 thousand. In the following surveys, there was a gradual decrease in the number of people living in the countryside and an increase in cities. This shows that at the beginning of the pandemic, students traveled from larger towns to the countryside. However, with the extension of this period, they slowly returned to the cities, which is possibly caused by the need for education in the health care units that are located there.

### 4.2 The impact of the pandemic on mental health

As the pandemic continued, studies showed the impact of the pandemic on mental health. Many authors highlighted newly emerged negative effects such as post-traumatic stress symptoms, confusion, and anger (48). An Egyptian online study showed that only about half of the respondents felt satisfied with their lives, whilst more people experienced severe psychological distress (49). The survey, published in The Lancet investigating the first 20 weeks of lockdown in England gave interesting results. The authors highlight that 12% of respondents had a score indicating moderate anxiety 10% had a score indicating severe anxiety, while 53% of participants had a score suggesting minimal anxiety. On the other hand, 26% of them had a score indicating at least moderate depression. However, after 20 weeks, there was a significant decrease in both anxiety and depressive symptoms, but being younger or living alone were all risk factors for higher levels of anxiety and depression at the start of the lockdown (50). Additionally, a survey from China identified a major mental health burden of the public during the COVID-19 pandemic. Younger people spent too much time thinking about the pandemic and healthcare workers were at high risk of mental illness. What is more, the prevalence of generalized anxiety disorders and depressive symptoms of the public were 35.1% and 20.1%, respectively (51). When it comes to students the results are worthy of attention. A study from the US points out that college students have experienced heightened stressors and reported stress-related drinking. Moreover, participants experienced mid-range levels of general stress and COVID-specific stress (52). The data from the British university showed that 84.2% of healthcare-related students reported worrying too much about different things and 61.9% could not stop or control worrying. Moreover, 72.1% of them felt unable to cope with their tasks at least sometimes and 8.5% never, or almost never, felt confident about handling personal problems (53).

The results from our study present significant differences in the well-being of the respondents and seem to confirm the mentioned works. This is also consistent with our working hypothesis—that the pandemic had a negative impact on the well-being of the study group. Moreover, there seems to be a clear trend, consistent with the development of the pandemic, i.e. the uncertainty of its consequences and legal restrictions. In T2 we recorded a large number of respondents experiencing “anxiety or fear for themselves or their families” – compared to 6 times less in T3 and 2 times less than in T4. Moreover, 17.89% of respondents felt “more stressed”, whilst in the following periods the percentage of these students was lower by about 30–40%. Worth mentioning is the fact that the largest number of people “feeling lonely” in T3, i.e. when the universities transitioned again to remote classes. As the pandemic continued, the percentage of students who were negatively affected increased, and the percentage of students who were positively affected or those without opinion clearly diminished (resulting in a lack of positively affected during the last measurement). Comparing this to the papers mentioned above, our results are similar to those from England (50). In the beginning, we also obtained a high level of anxiety, which in subsequent studies was 2–3 times lower. Similarly, the percentage of students who are “more stressed” decreased. When it comes to our study, the highest measurement of stress was noted in T2. This may be due to the fact that people were concerned about the lack of certain information on how the situation would develop further and how long it may last. The subsequent periods show a marked decline, probably due to the apparent trend toward a slow end to the pandemic. Other factors may be the previously mentioned negative emotions resulting from the return to classes and the worse autumn weather. There are similar changes in anxiety. However, it was declared by far fewer respondents in T3—perhaps less influenced by certain variables that caused students to declare themselves to feel more stressed. It would be necessary to conduct a study in which these variables are juxtaposed along with stress levels to determine possible correlations that could serve for more accurate interpretation in future studies. In addition, compared to the research from the British university, the percentage of students experiencing anxiety at our university was significantly lower—about 3–6 times, depending on the period (53). A noteworthy observation is the rising trend of people claiming to feel the same as before the pandemic. This possibly indicates that well-being is slowly returning to its pre-pandemic state as a result of the reduction of legal restrictions and morbidity.

### 4.3 Physical activity, exercise barriers, and motives

Another aspect of life affected by the COVID-19 pandemic was physical activity. In Poland, the first lockdown in March 2020 resulted in the confinement to homes, cancellation of competitions, and closure of sports facilities. Remote learning and work became the norm. The subsequent wave in October 2021 brought back similar restrictions, except for a national lockdown. Similar types of restrictions were also introduced by other countries, which changed the lifestyle of people and their daily activities (54, 55).

Although population studies conducted in various countries indicate changes in the physical activity of societies, they do not show their direct direction. A study from China shows that not only has the health-promoting lifestyle score increased, but also the level of exercise (56). On the other hand, a survey from Chile shows that more than 58% of respondents were sedentary during the pandemic, and slightly fewer were sedentary for 6 hours or more (43). In addition, research in Spain shows that the percentage of people exercising 1–3 and 4–5 times a week decreased, and that the percentage of people exercising 6 or more and not at all increased (42).

When it comes to studies among students, most indicate a decline in physical activity. Sekulic et al. in their work showed a significant increase in the average time spent sitting. In addition, during the lockdown, the most common activity shifted from walking to exercising at home. On the other hand, walking decreased from 40.7 to 24.2% of respondents during the pandemic (57). According to data obtained at a US university, there was a significant decrease in the use of gyms, the student recreation center, outdoor activities, and running outside and inside. Moreover, overall levels of physical activity decreased. There was an increase in moderate activity, but also a significant decrease in high activity and an increase in low activity. On the other hand, there was a significant increase in the use of self-created and guided at-home workouts, as well as the use of workout apps (58). Similar results were also obtained in Taiwan. During online learning, the level of physical activity of the students participating in this study was lower. Compared to the pre-pandemic period, participants were significantly physically inactive and spent 50% less time exercising (59). The study conducted in Great Britain also shows that 45.5% of participants exercised less (53). On the other hand, Romero-Blanco et al. observed a significant increase in the number of days on which students engaged in physical activity, as well as the total number of minutes of physical activity per week. Moreover, during COVID-19 pandemic daily sitting time also increased by 141.67 min (31).

Our research carried out on nursing students in Wrocław, shows a similar trend as research conducted at other universities. Although in our own study, conducted on medical students, we did not obtain statistically significant results due to the low level of activity already before the COVID-19 pandemic, we now recorded significant changes caused by it (60). Therefore, the working hypothesis about the negative pandemic impact on the physical activity of students is confirmed. The highest physical activity was observed in T0, i.e. before the start of the pandemic. In subsequent measurements there was a systematic decline in the IPAQ level. Disturbingly low results were noted in T4, when a low level of physical activity was obtained by over 86% of respondents. When it comes to MET, the highest average was also in T0 (before the pandemic in Poland), but the lowest average was in T1. However, there was an increase in T2, we noted a systematic decrease in subsequent surveys as with the level of IPAQ. In addition, we obtained similar results in the time spent walking as the research from Serbia (57). The highest average was before the pandemic in T0−125.1 minutes, and the lowest average in T1−29.1 minutes. Interesting results were noted in the time spent sitting. The lowest average was in T1- at the start of the pandemic. This may be due to the dynamic movement from Wrocław to the hometowns of respondents.

The most common exercise barriers were “lack of motivation” and “lack of time”. No barriers were reported from 2.1% in T2 to 25.2% of respondents in T1 (lockdown). Moreover, “lack of time” was extremely low in T1 (3.9%). This may be due to the increased free time caused by canceled classes for two weeks at our university. In addition, in T1 “lack of appropriate equipment” (28.2%), “applicable state regulations” (35.9%), and no “possibility of using favorite forms at home” (35.9%) were much more common, which was most likely caused by the national lockdown and the closure of sport facilities. On the other hand, the most common motives were: “improving physical fitness” (66%), “aesthetic goals” (62%), “improving or maintaining health” (46%). “The fight against depression” from T0 to T3 oscillates between 8–10%. It is worth noting that in T4 it is as much as 21.8%. This may be due to the negative impact of the pandemic on our respondents.

### 4.4 Alcohol and cigarettes

Alcohol and nicotine, widely used stimulants, contribute to millions of deaths annually. Moreover, their consumption may impact the incidence and progression of COVID-19 by weakening immunity and increasing susceptibility to viral infection. Alcohol and nicotine abuse can lead to health complications and chronic diseases. These substances are often used as coping mechanisms to avoid problems and manage stress. In contrast, during the pandemic, prolonged periods of staying at home heightened tension and stress, as observed in our study (61–64).

Much research has been done on the impact of the pandemic on drinking and smoking, indicating changes during this period. Sidor and Rzymski recorded an increase in alcohol consumption in nearly 15% of respondents and point out that some individuals may be more prone to alcohol overuse (41). A study from Australia shows an even higher percentage of respondents who saw an increase in alcohol consumption of 20% and the need to create a campaign on alcohol during the pandemic (65). The highest percentage of respondents with an increase in consumption was reported in Germany−35.5%, and the authors also encourage information campaigns (66). On the other hand, a study in Brazil reports that more than 20% of respondents have no or less alcohol consumption, with an increase of only 7.5% (67). Our previous study on medical students in Wrocław is similar, when we obtained an average decrease in alcohol consumption by 20% (60). In contrast, the results from the current nursing study show an initial decline and lowest risk of alcohol dependence in T1 compared to T0. However, in subsequent studies in T2 and T4, we saw a decrease in the percentage of people having a low level of risk and an increase in the number of people who consume alcohol at risk. It is also worth noting the increase in the percentage of people with a low level of risk of addiction in T3 and the fact that average alcohol addiction in studies was conducted in October was 2–4 times higher than those performed in March. This may be due to an increase in the social life of students at the start of the academic year. However, comparing the pre-pandemic period with October 2021, i.e. the most difficult pandemic period in Poland, with the highest average level of alcohol addiction among our students, this level increased by over 30% on average. This shows that despite the initial drop in addiction in the beginning, there was a high rebound, comparable to that in Germany at the beginning of the pandemic. Therefore, the working hypothesis about the negative impact of the pandemic on the alcohol addiction of students is confirmed. Going forward, we checked if there was a connection between alcohol addiction and the level of physical activity, but the results were not statistically important (Table 6).

When it comes to smoking, we did not note statistically significant changes therefore, we reject our working hypothesis of the negative impact of the COVID-19 pandemic on nicotine addiction. Although our study found no significant changes, other authors had important results. Studies from Germany and Poland showed that the percentage of people who started smoking more during the pandemic is in the majority, around 45% (41, 66). On the other hand, authors from Brazil and Italy recorded a lower percentage −2.6% and 9.1%, respectively (67, 68). In addition, Koczkodaj et al. point out that smokers are receptive to information about the COVID-19 pandemic and the risks of smoking. Moreover, mass, informational campaigns can help in quitting smoking (69).

## 5 Conclusions

The COVID-19 pandemic had a serious impact on the daily lives of the students. Weight fluctuations (*p* = 0.467) and BMI values (*p* = 0.558) were sinusoidal and there was no clear trend. As the pandemic continued, the percentage of students who were negatively affected increased and the percentage of students who were positively affected decreased to zero (*p* = 0.064). We recorded a large number of respondents experiencing anxiety / fear for themselves or their families, and also being more stressed in October 2020. In the later period, these numbers decreased, while the percentage of respondents who felt as before increased (*p* = 0.021). The highest physical activity was observed in the first measurement, i.e. before the start of the pandemic, and the lowest in the last measurement. Between these measurements, there was a systematic decline in the IPAQ level (*p* < 0.001). The most common exercise barriers (*p* < 0.001) were lack of motivation and lack of time. On the other hand, the most common motives (*p* < 0.001) were: improvement in physical function, aesthetic values, improving or maintaining health, and maintaining physical fitness. The highest average level of alcohol addiction (*p* < 0.001) was in October 2021 (8.71), as with nicotine addiction (*p* = 0.224). Moreover, risky alcohol consumption was significantly the most frequent in October 2021 and despite the initial drop in addiction in the beginning, there was a high rebound.

## 6 Limitations of the study

Despite conducting a comprehensive study on the impact of the COVID-19 pandemic, our work has limitations. The research group consisted mainly of nursing students, representing only a small fraction of the Polish population and the university community. As a result, gender-specific analysis could not be performed due to the low number of male participants. Additionally, the absence of individuals with chronic diseases or mental disorders among our respondents may limit the generalizability of the results. Biometric data was declared rather than measured due to COVID-19 restrictions, and nutrition status was not assessed, which could impact lifestyle and quality of life. In conclusion, expanding the study and increasing the number of participants would be necessary before extrapolating the impact of the pandemic to the wider adult population.

## Data availability statement

The raw data supporting the conclusions of this article will be made available by the authors, without undue reservation.

## Ethics statement

The studies involving humans were approved by the Institutional Review Board (or Ethics Committee) of the Bioethics Committee at the Medical University of Wroclaw (No. KB-251/2020). The studies were conducted in accordance with the local legislation and institutional requirements. The participants provided their written informed consent to participate in this study. Written informed consent was obtained from the individual(s) for the publication of any potentially identifiable images or data included in this article.

## Author contributions

Conceptualization and resources: AK. Data curation and methodology: AK, PK, and MW. Formal analysis: PK and MW. Supervision, visualization, and writing—review and editing: AK, PK, MW, and BA. Writing—original draft: AK, PK, MW, and ZK. All authors contributed to the article and approved the submitted version.
